# Regulation of specialised metabolites in Actinobacteria – expanding the paradigms

**DOI:** 10.1111/1758-2229.12629

**Published:** 2018-04-06

**Authors:** Paul A. Hoskisson, Lorena T. Fernández‐Martínez

**Affiliations:** ^1^ Strathclyde Institute of Pharmacy and Biomedical Sciences University of Strathclyde, 161 Cathedral Street Glasgow G4 0RE UK; ^2^ Department of Biology Edge Hill University, St Helens Road Ormskirk Lancashire L39 4QP UK

## Abstract

The increase in availability of actinobacterial whole genome sequences has revealed huge numbers of specialised metabolite biosynthetic gene clusters, encoding a range of bioactive molecules such as antibiotics, antifungals, immunosuppressives and anticancer agents. Yet the majority of these clusters are not expressed under standard laboratory conditions in rich media. Emerging data from studies of specialised metabolite biosynthesis suggest that the diversity of regulatory mechanisms is greater than previously thought and these act at multiple levels, through a range of signals such as nutrient limitation, intercellular signalling and competition with other organisms. Understanding the regulation and environmental cues that lead to the production of these compounds allows us to identify the role that these compounds play in their natural habitat as well as provide tools to exploit this untapped source of specialised metabolites for therapeutic uses. Here, we provide an overview of novel regulatory mechanisms that act in physiological, global and cluster‐specific regulatory manners on biosynthetic pathways in Actinobacteria and consider these alongside their ecological and evolutionary implications.

## Introduction

Actinobacteria exhibit staggering diversity in terms of their biosynthetic capability for specialised metabolites such as antibiotics, antifungals, antihelminthics, antivirals and immunosuppressives. The production of specialised metabolites requires the integration of a range of environmental and physiological inputs to ensure appropriate production. The conventional view of these organisms was that each species was capable of producing one or two different specialised metabolites from clusters of coordinately regulated genes on the chromosome. However, genome sequencing efforts have revealed that there are a large number of specialised metabolite biosynthetic gene clusters (BGCs) in actinobacterial genomes that encode for the production of potentially useful metabolites which are not actively expressed under commonly used laboratory conditions. Prior to the sequencing of the first *Streptomyces* genome sequence, the genus was estimated to have the potential to produce in the region of 150,000 bioactive compounds (Watve *et al*., 2001). The availability of numerous *Streptomyces* genome sequences now suggests that this is a gross underestimation of their biosynthetic capabilities and that there is still a large reservoir of untapped bioactive molecules to be discovered from these organisms. These BGCs are the so called ‘cryptic biosynthetic pathways’, and exploiting these will require a thorough understanding of the environmental cues and regulatory mechanisms that trigger the production of these specialised metabolites.

**Figure 1:**
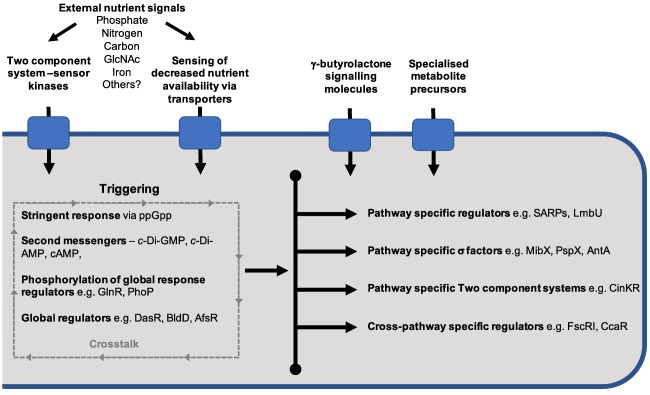
Schematic summarising the complex range of external and internal factors influencing the regulation of specialised metabolite production in Actinobacteria particularly focusing on the novel mechanisms discussed in this review. A range of external signals can trigger specialised metabolite production either through the direct activation of pathway specific regulators or indirectly via an intricate network of global regulators and intracellular signalling molecules. The signalling molecules and other regulatory elements contained in the dotted box are also subject to complex interactions amongst themselves. The outcome of these complex interactions will determine the activation or repression of secondary metabolite biosynthetic gene clusters.

The majority of Actinobacteria that have extensive specialised metabolism inhabit competitive and nutrient limited environments such as soil and sediment, and these highly structured and dynamic locations comprised a multitude of diverse microniches in which taxonomically diverse organisms reside and compete for resources (Stubbendieck *et al*., 2016; Behie *et al*., 2017). It is a competition in these environments that likely drive the evolution of these adaptive responses (Vargas‐Bautista *et al*., 2014; Traxler *et al*., 2015; Behie *et al*., 2017). Key to the adaptive nature of production is the appropriate and coordinated production of these chemically diverse compounds, the regulation of which has been the focus of specialised metabolite research for many years. The identification of a range of regulatory mechanisms indicates that highly elaborate regulatory mechanisms act at all levels of expression from transcriptional control through to translational control mechanisms (Chandra and Chater, 2008). Early work on the direct control of specialised metabolites identified the pathway‐specific regulator proteins [also known as Cluster Situated Regulators (CSR); Huang *et al*., 2005] such as ActII‐ORF4, RedD and CdaR (White and Bibb, 1997; Wietzorrek and Bibb, 1997; Ryding *et al*., 2002), which were amongst the first members of the well‐studied *Streptomyces* antibiotic regulatory protein (SARP) protein family to be identified (Wietzorrek and Bibb, 1997). Later, the role of γ‐butyrolactones was identified as an extracellular signal for the induction of specialised metabolites (Horinouchi and Beppu, 1994; Hsiao *et al*., 2007), which were later shown to act through cytoplasmic binding proteins to activate CSRs in a range of Actinobacteria (Sidda and Corre, 2012). The identification of pleiotropic regulators of specialised metabolites such as the AfsR (Floriano and Bibb, 1996) and the AbsA1A2 two component system in the well‐studied strain *Streptomyces coelicolor* (Hutchings *et al*., 2004; Huang *et al*., 2005) indicated that there is a complex multilevel control of biosynthesis. This also highlighted that these organisms must integrate a number of signals to regulate specialised metabolite biosynthesis in an appropriate manner (Horinouchi *et al*., 1990; Floriano and Bibb, 1996; Horinouchi, 2003; Parajuli *et al*., 2005; Maharjan *et al*., 2009). This work has been reviewed extensively by Bibb (2005), Van Wezel and Mcdowall (2011) and Liu and colleagues (2013); however, recent work has added greater detail and new mechanisms of regulation have emerged. Here, we discuss some new regulatory mechanisms that add to our burgeoning knowledge of environmental signal integration and how this affects the regulation of specialised metabolites. Figure 1 illustrates the complex range of external and internal factors influencing the regulation of specialised metabolite production in Actinobacteria, particularly focussing on the novel mechanisms discussed in this review.

## Physiological regulation – integrating starvation signals in to specialised metabolite biosynthesis

The production of specialised metabolites in actinomycetes is tightly regulated. Direct regulation occurs at the level of the BGC, but it is often in response to the producing organism responding to their complex and changing environment. This is unsurprising as laboratory studies have shown that the activation of these pathways is often at the point when nutrients are becoming depleted. This offers significant challenges to the primary metabolism of producing organisms to cope with maintenance of cellular function and production of costly specialised metabolites (Schniete *et al*., 2018). Integrating physiological signals into the production of specialised metabolites ensures that these are only produced when required and this process is usually coordinated with the onset of morphological differentiation (Bibb, 2013), thus optimising the use of energy and resources.

For instance, DasR, a GntR‐like repressor, responds to environmental conditions by sensing the levels of the chitin monomer GlcNAc and integrates the regulation of primary metabolism, development and antibiotic production in Actinobacteria. The role of DasR in the genus *Streptomyces* has been recently reviewed in Urem and colleagues (2016). Inorganic phosphate starvation also triggers the production of several specialised metabolites in actinomycetes (Nieselt *et al*., 2010; Allenby *et al*., 2012; Martín *et al*., 2017). This activation is in many instances indirectly mediated by the master response regulator PhoP via signal transduction cascades, which ultimately activate pathway‐specific regulators (Rodríguez‐García *et al*., 2007). Similarly, GlnR, the master response regulator for nitrogen metabolism in actinomycetes, which also plays an important role in carbon uptake and metabolism in these organisms (Liao *et al*., 2015), has been recently found to directly activate pathway‐specific regulators of specialised metabolites in several actinomycete genera (Yao *et al*., 2014; He *et al*., 2016).

It is important to note that the above global physiological master regulators are subject to intricate cross‐regulatory metabolic networks, which have an effect on the production of specialised metabolites in actinomycetes as recently reviewed by Urem and colleagues (2016).

A link between guanosine tetraphosphate (ppGpp) synthesis and the regulation of antibiotic production in actinomycetes has long been established. ppGpp is a key intracellular signalling molecule, which links nutrient starvation sensing with adaptive responses in a wide range of bacteria including actinomycetes. Under conditions of nitrogen limitation, ppGpp is synthesised by the ribosome bound protein RelA (Cashel *et al*., 1996). Although ppGpp synthesis has been previously shown to be required for antibiotic production under conditions of nitrogen limitation at least in *S. coelicolor* (Chakraburtty and Bibb, 1997; Hesketh *et al*., 2001), no evidence of the direct effect of ppGpp synthesis on the regulation of a specialised metabolite biosynthetic pathway had been provided until recently.

In the soil actinomycete *Microbispora corallina*, ppGpp synthesis has been shown to activate the complex regulatory pathway that leads to the biosynthesis of microbisporicin, a potent lantibiotic currently undergoing preclinical trials. ppGpp is responsible for the activation of transcription of *mibR*, encoding for one of the three regulators in the microbisporicin gene cluster. MibR in turn leads to the production of a precursor, which subsequently induces high levels of production of the mature antibiotic in a feed‐forward regulatory mechanism (Fernández‐Martínez *et al*., 2015). While microbisporicin is the only confirmed example of the direct effect of ppGpp over the regulation of specialised metabolite biosynthesis, it is speculated that this newly identified physiological regulatory mechanism is common amongst other actinomycetes including the planosporicin producer *Planomonospora alba* (Sherwood and Bibb, 2013).

Cross‐regulation of biosynthetic gene clusters encoding for specialised metabolites can also occur when they share precursors. Secondary metabolism is dependent on the availability of precursors from primary metabolism. Primary metabolites from the central carbon metabolism, which function as building precursors for secondary metabolites, include oxaloacetate, α‐ketoglutarate, glyceraldehyde‐3‐phosphate, glucose‐6‐phosphate and acetyl‐CoA (Huang *et al*., 2015; Hiltner *et al*., 2015). For example, acetyl‐CoA is a precursor for polyketide biosynthesis, which can be generated via degradation of triacylglycerols. Thus, actinomycete species, which degrade storage lipids at a higher rate, will provide higher levels of acetyl‐CoA and ultimately produce higher levels of specialised polyketide metabolites (Le Maréchal *et al*., 2013) and hence why oil‐based fermentation media are used extensively for industrial polyketide biosynthesis (Hiltner *et al*., 2015). When more than one polyketide is encoded in the genome of the same strain, it is likely that one of these metabolites will be produced preferentially. Yang and colleagues (2017) have recently reported that although spirotoamides and tautomycetin (TTN) are compounds synthesised by two distinct type I PKSs, competition for the same pool of acyl‐CoA precursors results in the preferential production of TTN in *Streptomyces griseochromogenes*. Deletion of the positive regulators involved in TTN biosynthesis liberates precursors for the production of spirotoamides. Hence understanding how precursors are shared by different biosynthetic clusters may lead to the generation of strains, which produce previously undetectable compounds.

## Global regulation – coordinating regulation across the genome and across pathways

Originally identified as a highly conserved regulator of aerial mycelium formation and of antibiotic production in *S. coelicolor*, the autoregulatory protein BldD has been shown to play a central role in regulating sporulation and specialised metabolite production in *Streptomyces* (Elliot *et al*., 1998; 2001; Elliot and Leskiw, 1999; Den Hengst *et al*., 2010; Al‐Bassam *et al*., 2014). BldD was recently shown to act through the binding of the second messenger *c‐di‐*GMP, expanding the number of known molecules that affect specialised metabolite expression. In *S. coelicolor* and *Streptomyces venezuelae*, BldD directly represses genes involved in sporulation, indirectly affecting specialised metabolite production through the regulation of *bldA*; however, direct control of erythromycin production in *Saccharopolyspora erythrea* and the SapB‐like lantipeptide AmfS in *Streptomyces griseus* has been demonstrated (Ueda *et al*., 2005; Chng *et al*., 2008). It remains to be seen how common second messenger regulation of specialised metabolites is within streptomycetes; however, emerging work is expanding the roles for other secondary messengers in *Streptomyces* such as cAMP in leinamycin biosynthesis and cyclic‐di‐AMP control of muralytic enzymes (St‐Onge and Elliot, 2015; Huang *et al*., 2016; Tschowri, 2016; St‐Onge *et al*., 2017), suggesting that this will be a future area of focus in the study of these organisms.

Two component regulatory systems are amongst a plethora of global regulators that affect transcriptional responses in streptomycetes, including the production of specialised metabolites. The highly conserved MtrAB two component system has long been known to be an important global regulator in Actinobacteria, coordinating a range of cellular responses and essential cell division processes such as DnaA, FtsI, DivIVA and FtsZ in *Corynebacterium*, *Mycobacterium* and *Streptomyces* (Via *et al*., 1996; Möker *et al*., 2004; Cangelosi *et al*., 2006; Hoskisson and Hutchings, 2006; Clark *et al*., 2013). Recently, the repertoire of genes controlled by MtrAB was expanded to include the BGC for the antibiotic chloramphenicol in *S. venezuelae* (Som *et al*., 2017a). Using ChIP‐seq analysis of MtrA, Som and colleagues (2017a) demonstrated direct binding of the response regulator MtrA to genes of the chloramphenicol as well as sharing target genes with other Actinobacteria (such as *dnaA*, *dnaN*, *oriC* and *wblE* in *Mycobacterium*). Moreover, MtrA binds to the promoters of the streptomycetes‐specific cell division genes *ssgA* and *ssgB* (Som *et al*., 2017a). Interestingly, deleting the sensor kinase, MtrB resulted in constitutive chloramphenicol production and a global shift in the metabolome of *S. venezuelae*. Expanding these studies to *S. coelicolor*, the same authors were able to show direct binding of MtrA upstream of the CSRs *actII‐Orf4* and *redZ*, showing direct regulation of actinorhodin and undecylprodigiosin respectively (Som *et al*., 2017b). These data suggest that deletion of the sensor kinase MtrB uncouples the control of morphological and physiological differentiation in streptomycetes, perhaps through mimicking of an as yet undetermined environmental signal resulting in the upregulation of BGCs. This suggests that manipulation of the MtrAB system could be exploited as a universal process to unlock the biosynthetic potential of these organisms through the activation of cryptic biosynthetic pathways as has also been shown for the AfsQ system (Daniel‐Ivad *et al*., 2017). The role played by a range of two component regulatory systems in specialised metabolism has expanded enormously in recent years. Systems such as AfsQ1/Q2 (Ishizuka *et al*., 1992) and AbsA1/A2 (Anderson *et al*., 2001) have been shown to have direct interactions with metabolic responsive regulators such as GlnR (Wang *et al*., 2013) and PhoP (Santos‐Beneit *et al*., 2009; Yao and Ye, 2016) and can form complete nutrient‐sensing specialised metabolite activating signal transduction pathways (Yao and Ye, 2016). This suggests that there is still much to be learned about interplay between the complex regulatory pathways of specialised metabolites and the role of two component regulators in these signal transduction pathways.

The genome sequences of Actinobacteria have revealed a huge and untapped resource of gene clusters encoding potentially bioactive molecules from a diverse range of chemical families. Intriguingly, their presence raises several questions around how these metabolites are cordinately expressed, given the potential for synergistic and antagonistic activities, and how they may be activated in response to extracellular and intracellular signals. Whilst the existence of pleiotropic regulators affecting expression of multiple BGCs is well known (Huang *et al*., 2005), cross‐regulation between clusters has rarely been shown. In *Streptomyces clavuligerus*, the β‐lactam antibiotic, cephamycin and the β‐lactamase inhibitor, clavulanic acid, are encoded contiguously on the genome in a ‘supercluster’ (Ward and Hodgson, 1993). Their biosynthesis is controlled in a coordinated manner by the SARP, CcaR located in the cephamycin BGC (Pérez‐Llarena *et al*., 1997; Alexander and Jensen, 1998; Santamarta *et al*., 2011). Interestingly, these molecules have complementary biological activities and may reflect the evolutionary conservation of their linkage in a ‘supercluster’. A recent article by McLean and colleagues (2016) showed coordinate regulation of two chemically unrelated, nonsynergistic specialised metabolites – antimycin and candicidin. The BGCs for both metabolites are separated by 9 kb on the chromosome of *S. albus* S4, but the authors were able to show that there were conserved binding sites for the pathways specific regulator of the candicidin BGC, FscRI directly upstream of the genes encoding the depsipeptide natural product antimycin (McLean *et al*., 2016). FscRI is not a SARP protein but a PAS‐LuxR family regulator, yet again extending the paradigm of activator proteins involved in specialised metabolite biosynthesis; moreover, one that directly regulates two different natural products. These data have implications for the production of heterologous BGCs in streptomycetes. McLean and colleagues (2016) showed that heterologous production of antimycin was only possible when *fscRI* was present in *trans*, implying that the simple approach of cloning cryptic BGCs for streptomycetes and expressing the BGC in a heterologous host such as *S. coelicolor* (Gomez‐Escribano and Bibb, 2011) may not be as straightforward as first thought and CSRs from other specialised metabolites may be required for expression.

## Cluster‐specific regulation – ECFs as new mechanisms of cluster specific regulation

Cluster‐specific regulation refers to regulatory elements dedicated to the transcriptional control (either via activation or via repression) of the particular biosynthetic gene cluster in which they are located. Traditionally in actinomycetes, this type of regulation is conducted by either transcriptional activators/repressors or two component regulatory systems (Van Wezel and Mcdowall, 2011; Liu *et al*., 2013) normally as part of a signalling cascade triggered by global or physiological regulators.

The extracytoplasmic function (ECF) sigma factors are a group of small regulatory proteins normally bound (when inactive) to a cognate membrane‐associated anti‐sigma factor. Once the appropriate inducing signal is detected, the anti‐sigma factor is inactivated either through modifications, conformational changes or proteolysis. This inactivation leads to the release of the ECF and enables it to be recruited by the RNA polymerase core enzyme, therefore allowing transcription initiation from ECF‐specific target promoters. Actinomycetes contain a large number of ECF sigma factors within their genomes compared to other bacterial groups (Staroń *et al*., 2009; Huang *et al*., 2015), thus highlighting the genetic and phenotypic plasticity of these organisms in terms of survival in complex and changing environments. ECF sigma factors are usually involved in the regulation of a wide range of physiological processes such as transport, secretion and extracytoplasmic and cytoplasmic stress response (Helmann, 2002). However, identification of several new biosynthetic clusters from different Actinobacteria has revealed the presence of ECF sigma/anti‐sigma pairs located within BGCs. Confirmation of their role as cluster‐specific regulators of specialised metabolites has been recently confirmed.

Antimycins are a family of depsipeptides produced by a hybrid nonribosomal peptide synthetase/polyketide synthase (NRPS)/PKS) assembly line. The *ant* biosynthetic cluster is present in at least 14 species of *Streptomyces* (Seipke and Hutchings, 2013), and an orphan ECF sigma factor, that is, it has no co‐encoded anti‐sigma factor, is conserved in all the *ant* clusters. In *S. albus* S4, two of the four operons involved in antimycin production are directly regulated by the orphan ECF sigma factor AntA (Seipke *et al*., 2014).

Cluster‐specific ECF sigma factor regulation has also been established in ribosomally synthesized and post‐translationally modified peptides (RiPPs). For example, the production of a much less active precursor form of the lantibiotic microbisporicin in *M. corallina* is initiated by ppGpp synthesis (see above). This precursor is then exported, and once outside the cell, it triggers the release of the cluster specific ECF sigma factor MibX from the membrane‐associated anti‐sigma factor MibW. MibX can now activate transcription of the whole biosynthetic cluster to reach high levels of production of the fully modified and active form of microbisporicin (Foulston and Bibb, 2011, 2010; Fernández‐Martínez *et al*., 2015). Similarly, production of the lantibiotic planosporicin in *P. alba* is controlled by a cluster‐specific ECF sigma factor (PspX). In this case, planosporicin itself is responsible for the release of the sigma factor to activate transcription of the biosynthetic cluster (Sherwood and Bibb, 2013). High levels of production of cinnamycin, a lantibiotic produced by *Streptomyces cinnamoneus*, are triggered by low levels of extracellular cinnamycin, but in this instance, it is a two‐component regulatory system, CinKR, responsible for the transcriptional activation of the biosynthetic cluster (O'rourke *et al*., 2017). In all three above cases, this feed‐forward regulatory mechanism seems to function to ensure that high levels of production of these compounds only occurs when immunity is already established in the population. This newly discovered way of regulating antibiotic production is likely to serve to coordinate the production of the compound throughout the whole mycelial population with a dual role: production of ecologically effective levels of the specialised metabolite as well as ensuring immunity amongst the whole colony.

## Concluding remarks: ecological and evolutionary implications of regulation

Given that the specialised metabolite producing Actinobacteria are a widely distributed group of organisms that occupy a wide range of environmental niches with an enormous repertoire of specialised metabolites, it is unsurprising that they have also evolved a vast array of mechanisms with which to coordinate and regulate the expression of molecules with adaptive function. The biosynthesis of specialised metabolites clearly has a larger evolutionary cost to the producing strain than carrying the resistance mechanism to a specialised metabolite where tens of kilobases of DNA are required to encode BGCs whereas only hundreds of bases are required for many resistance mechanisms (Traxler *et al*., 2015). Therefore, the energetic cost to produce the mRNA, translate the protein and utilise the primary metabolic building blocks to produce even the most modest chemical scaffold of a specialised metabolite must be large, especially in an environment where nutrients are becoming limited. The evolutionary strategy that these organisms have evolved is to tightly regulate and coordinate production of these costly metabolites to ensure that their production is timely and appropriate. Moreover, the incorporation of systems for the synchronisation of immunity mechanisms to ensure protection of the whole multicellular population is now being identified with increasing frequency. The discovery of the enormous array of signalling molecules, regulatory proteins, regulatory networks and pathways in Actinobacteria has shown that evolution has solved the problem in many different ways, and undoubtedly, there are novel mechanisms still to be elucidated, such as the recently discovered novel LmbU family of CSRs, which appears to be widespread in BGCs of Actinobacteria (Hou *et al*., 2018).
